# Genetic and physical interaction of Ssp1 CaMKK and Rad24 14-3-3 during low pH and osmotic stress in fission yeast

**DOI:** 10.1098/rsob.130127

**Published:** 2014-01-22

**Authors:** Silja I. Freitag, Jimson Wong, Paul G. Young

**Affiliations:** 1 Department of Biology, Queen's University, 116 Barrie Street, Kingston, Ontario, CanadaK7L 3N6; 2 Department of Biological Sciences, Purdue University, 915 West State Street, West Lafayette, IN 47907, USA

**Keywords:** Ssp1, Rad24, 14-3-3, hyperosmotic stress, relocalization, pH

## Abstract

The Ssp1 calmodulin kinase kinase (CaMKK) is necessary for stress-induced re-organization of the actin cytoskeleton and initiation of growth at the new cell end following division in *Schizosaccharomyces pombe*. In addition, it regulates AMP-activated kinase and functions in low glucose tolerance. *ssp1^−^* cells undergo mitotic delay at elevated temperatures and G_2_ arrest in the presence of additional stressors. Following hyperosmotic stress, Ssp1-GFP forms transient foci which accumulate at the cell membrane and form a band around the cell circumference, but not co-localizing with actin patches. Hyperosmolarity-induced localization to the cell membrane occurs concomitantly with a reduction of its interaction with the 14-3-3 protein Rad24, but not Rad25 which remains bound to Ssp1. The loss of *rad24* in *ssp1^−^* cells reduces the severity of hyperosmotic stress response and relieves mitotic delay. Conversely, overexpression of *rad24* exacerbates stress response and concomitant cell elongation. *rad24^−^* does not impair stress-induced localization of Ssp1 to the cell membrane, however this response is almost completely absent in cells overexpressing *rad24*.

## 2. Introduction

Environmental stress causes massive changes to cell physiology, including major metabolic, cytoskeletal and transcriptional responses. Changes in osmolarity, temperature, reactive oxygen species and nutrient status stimulate conserved stress-activated Spc1 MAPK pathways [1–5]. In fission yeast, the Wak1 and Win1 [6,7], Wis1 [8,9] and Spc1 MAPK cascade [9,10] relays environmental signals to the nucleus. Phosphorylated Spc1 MAPK enters the nucleus and activates transcription factors, inducing stress response genes including *gpd1* (glycerol-3-phosphate dehydrogenase) and *tps1* (trehalose-6-P synthase), increasing intracellular concentrations of glycerol and trehalose [10–13]. MAPK signalling impinges on the cell cycle via Srk1, which phosphorylates the mitotic activator Cdc25, inducing 14-3-3 dimer binding and nuclear export of Cdc25 thus reducing the opportunity to activate its CDK nuclear substrate, Cdc2 [14]. *spc1*^−^ cells experience G_2_ delay under normal conditions and G_2_ arrest after osmotic or oxidative stress [9,15,16].

The CaMKK and CaMKI and CaMKV calcium–calmodulin (Ca^2+^/CaM)-dependent signalling cascade is highly conserved and involved in a number of important cellular processes including cell cycle and neuronal- and immune-cell function. CaMKKs phosphorylate and fully activate Ca^2+^/CaM-bound CaMKs as well as protein kinase B and AMP-activated kinase (AMPK) [17]. CaMKK activity is regulated by activating and inhibitory phosphorylation as well as Ca^2+^/CaM binding. CaMKKβ retains 50–70% constitutive activity but requires Ca2^+^/CaM binding for full activation [18].

The fission yeast CaMKK orthologue Ssp1 [19] was identified [20] in a screen for suppressors of the cell morphology mutants *ppe1* [21] and *sts5–7* [22], and independently as a pH and temperature sensitive loss of function cell-cycle mutant [23]. Ssp1 phosphorylates Ssp2, the catalytic subunit of AMPK [24,25] and is required for efficient growth in low glucose conditions [19]. AMPKs regulate energy homoeostasis and respond to glucose [26], playing a role directly or indirectly in coupling nutritional response to cell differentiation in fission yeast [24]. In budding yeast, glucose depletion and environmental stressors lead to the activation of AMPK homologue SNF1 via SAK1, TOS3 or ELM1 kinases [27,28]. AMPK negatively regulates glycerol-3-phosphate dehydrogenases GPD1 and GPD2. GPD1 is inhibited in high glucose by TORC2-dependent kinases and AMPK and activated upon glucose limitation. Cells rapidly adapt to hypertonicity through a rapid increase in GPD1 activity via reduction of TOR2C-YPK1/2-mediated phosphorylation, and transcriptionally also upregulate GDP1 within 60 min. When glucose is restricted, AMPK inhibits GPD2 to limit glycerol production [29]. *ssp1^−^* has a pleiotropic phenotype and is synthetically lethal with *spc1^−^* under conditions permissible for either single mutant [23]. At high temperatures, *ssp1* mutants grow as monopolar cells with a reduced capacity for transient stress-activated dispersion of actin monomers, suggesting a role for Ssp1 in actin mobilization [20,23]. Loss of *ssp1* disturbs growth polarity and increases cell morphology aberrations, for example branching [30]. At high temperatures, in the presence of low pH (3.5) or hyperosmolarity (0.6 M KCl), *ssp1* mutants cannot proliferate; instead they complete DNA replication and arrest as highly elongated cells in G_2_ [20,23].

Although largely cytoplasmic in localization, several pools of Ssp1 exist in the cell, and following osmotic stress a portion localizes to the cell membrane or cortex. Here, we explore the physical interaction of the CaMKK Ssp1 with the 14-3-3 orthologues Rad24 and Rad25 and their relationship to the rapid movement of a portion of the Ssp1 cytoplasmic pool to the cell cortex following stress.

## 3. Results

### 3.1. *rad24* deletion suppresses the cell-cycle phenotype of *ssp1^−^* cells at high temperatures

**Figure 1.**
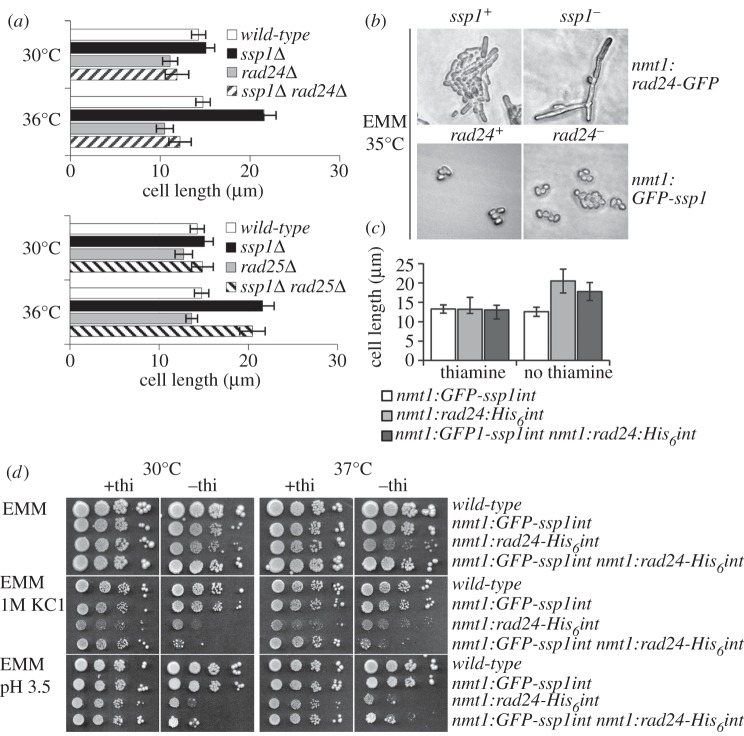
Effect of high temperature on *ssp1*^−^. (*a*) Suppression of cell-cycle delay in *ssp1^−^* by *rad24*^−^. Cells (YEA) were shifted from 30 to 36°C for 4 h. There is no significant difference in cell lengths of *ssp1*^−^ *rad24^−^* cells at 30 and 36°C (*p* = 0.198). There is a significant difference in cell lengths of *ssp1^−^ rad25^−^* cells at 30 and 36°C (*p* < 0. 05) (all *n* ≥ 37; all Student's *t*-test). (*b*) Cell-cycle and morphological effect of overproduction of Rad24 and Ssp1. Plasmid expression in cells was induced in cells growing for 20 h (25 or 35°C) on media lacking thiamine. (*c*) Cells (30°C, EMM + thiamine) were washed (EMM) and derepressed for 24 h. Cells overexpressing *GFP-ssp1int* are significantly shorter in the absence than in the presence of thiamine (*p* < 0. 05) (all *n* > 117; Student's *t*-test). (*d*) Cells (30°C, EMM + thiamine) were washed with EMM and diluted (10^6^, 10^5^, 10^4^ and 10^3^ cells ml^−1^). Five microlitres of each cell suspension were spotted onto media as indicated and incubated at 30 and 37°C for 5 days.

We identified the 14-3-3 homologues Rad24 and Rad25 [31] multiple times in a yeast two-hybrid screen using full-length Ssp1 as a bait protein (data not shown), corroborating previous mass spectrometry data [19]. 14-3-3 proteins inhibit CaMKKα in mammalian systems [32] and are directly linked to the control of cell-cycle progression by regulating the Cdc2/Cdc13 activator Cdc25 [33] and inhibitor Wee1 [34–36]. In fission yeast, neither 14-3-3 isoform is essential; however, the double deletion is lethal [31]. To test for the influence of Rad24 on the mitotic delay of *ssp1^−^* cells at high temperatures [20,23], *ssp1^−^ rad24^−^* (Q4101; table 1) and *ssp1^−^ rad25^−^* (Q4104) cells (YEA) were shifted from 30 to 36°C for 4 h (figure 1*a*). Loss of *rad24* is epistatic with respect to the heat-stress-dependent cell elongation phenotype of *ssp1^−^* cells at 36°C. Loss of Rad25 has no effect, presumably owing to the small proportion of the *rad25* 14-3-3 isoform in the overall pool of 14-3-3 proteins (see figure 7*d*).

**Table 1.** List of strains.

strain	genotype	source
Q1618	*URA4::lexAop-lacZ/8LEXA-ADE2::URA3 ura3-1/ura3-1 leu2-3/leu2-3 his 3-11/his3-11 trp1-1/trp1-1 ade2-1/ade2-1 can1-1/can1-1*	laboratory stock
Q250	wild-type *(972 h-)*	laboratory stock
Q3677	*leu1-32 ura4-D18*	laboratory stock
Q4101	*ssp1::ura4^+^ leu1-32 ura4-D18*	Toda laboratory
Q1537	*ssp1::sup3-5 ade-6-704 ura4-D18 leu1-32*	laboratory stock
Q4101	*ssp1::ura4^+^ rad24::ura4^+^ ura4-D18 leu1-32*	this study
Q4102	*rad24::ura4^+^ ura4-D18 leu1-32*	Carr laboratory
Q4103	*rad25::kanMX6 ura4-D18 leu1-32*	this study and Carr laboratory
Q4104	*ssp1::ura4^+^ rad25::kanMX6 ura4-D18 leu1*-32	this study
Q4105	*pREP1-rad24GFP* in *ssp1::sup3-5 ade6-704 leu1-32 ura4-D18*	this study
Q4106	*pREP1-rad24GFP* in *leu1-32 ura4-D18*	this study
Q4107	*pIR2-22* (*nmt1:GFP-ssp1*) in *leu1-32 ura4-D18*	this study and laboratory stock
Q4108	*pIR2-22* in *rad24 ::ura4^+^ ura4-D18 leu1-32 ade6-704*	lab stock
Q4109	*nmt1:rad24-His_6_int ura4-D18 leu1-32*	this study
Q4110	*nmt1:rad24-His_6_int nmt1:GFP-ssp1int ura4-D18 leu1-32*	this study
Q4111	*nmt1:GFP-ssp1int ura4-D18 leu1-32*	this study
Q2016	*cdc25-GFPint ura4-D18 leu1-32*	laboratory stock
Q4112	*cdc25-GFPint ssp1::sup3-5 ade6-704 ura4-D18 leu1-32*	this study
Q3974	*cdc25-GFPint rad24::ura4^+^ leu1-32 ura4-D18*	laboratory stock
Q4113	*cdc25-GFPint ssp1::sup3-5 rad24::ura4+ 5 ade6-704 ura4-D18 leu1-32*	this study
Q300	*cdc25-22 leu1-32 ura4-D18 ade6-10*	laboratory stock
Q1530	*cdc25-22 ssp1::sup3-5 ade6-704 ura4-D18 leu1-32*	laboratory stock
Q4114	*ssp1-GFPint ura4-D18 leu1-32*	this study
Q4115	*ssp1-GFPint rad25::ura4+ ura4-D18 leu1-32*	this study and Carr laboratory
Q4116	*ssp1-GFPint rad24 ::ura4+ ura4-D18 leu1-32*	this study and Carr laboratory
Q4117	*ssp1-CFPint arp3C-YFPint leu1-32 ura4-D18*	this study and Nolen
Q4118	*ssp1-GFPint rad24-2HA-His_6_*(*ura4^+^*)*int leu1-32 ura4-D18*	this study and Russell laboratory
Q4119	*ssp1-GFPint rad25-His_6_ ura4-D18 leu1-32*	this study
Q4120	*ssp1-GFPint rad24-2HA-His_6_*(*ura4^+^*)*int rad25-His_6_ leu1-32 ura4-D18*	this study and Russell laboratory
Q4121	*ssp1-GFPint rad25-His_6_ rad24::ura4^+^ ura4-D18 leu1-32*	this study
Q4122	*nmt1:GFP-ssp1int ura4-D18 leu1-32*	this study
Q4123	*ssp1-GFP:kanMX6int ura4-D18 leu1-32*	this study
Q4124	*pREP1-rad24-His_6_* in *ssp1-GFP:kanMX6int ura4-D18 leu1-32*	this study

### 3.2. Overproduction of Rad24 and Ssp1 results in an additive cell-cycle phenotype

Plasmid-borne *rad24-GFP* or *GFP-ssp1* (*pIR2-22*) [23], both under control of the strong thiamine-repressible *nmt1* promoter (*OP*-*rad24-GFP* and *OP-GFP-ssp1*), were expressed for 20 h in wild-type, *ssp1^−^* and *rad24^−^* cells, respectively (Q4105, Q4106, Q4107, Q4108) (figure 1*b*). In an otherwise wild-type background at 25°C, overproduction of Rad24 causes moderate cellular elongation, whereas overproduction of Ssp1 makes cells shorter [23]. Conversely, overproduction of Rad24 at 25°C in *ssp1^−^* cells leads to occasional branching, exacerbated at 35°C with extremely elongated cells often displaying aberrant branched morphology. The size reduction from *OP-GFP-ssp1* is more conspicuous in *rad24^−^* cells, which become spherical. Overexpression of *rad24* thus has an additive phenotype with *ssp1^−^*, exacerbating the cell elongation phenotype.

At 30°C, wild-type cells expressing a single chromosomally integrated copy of *rad24-His_6_* under control of the *nmt1* promoter (*OP*-*rad24-His_6_int*) (Q4109) are significantly longer (21.7±4.1 μm) than if expression is repressed (13.2±1.1 μm). By contrast, cells with single-copy expression of *GFP-ssp1* under control of the *nmt1* promoter (*OP*-*ssp1-GFPint*) (Q4111) are significantly shorter (12.9±1.0 μm) than if expression is repressed (13.9±1.2 μm). An intermediate size (19.2±2.4 μm) is found for cells co-overexpressing *ssp1* and *rad24* (Q4111), suggesting that these gene products antagonize each other in some way (figure 1*c*).

To compare growth rates, thiamine-repressed *OP-GFP-ssp1int* and/or *OP-rad24-His_6_int* cells were washed in EMM before plating diluted aliquots (EMM, EMM+ 1 M KCl and EMM pH 3.5; all ±thiamine) (figure 1*d*), followed by incubation for 5 days (30 or 37°C). At both temperatures, cells overexpressing *rad24* displayed inhibited growth compared with cells overexpressing *ssp1* or both *rad24* and *ssp1*. Overexpression of *rad24* negatively affects proliferation, resistance to hyperosmotic and low pH stress, and increases mitotic delay. Growth inhibition in *OP-rad24-His_6_int* cells was exacerbated by 1 M KCl (30 and 37°C) and *OP-GFP-ssp1int* did not alleviate this effect. Similar growth delay in *rad24* overexpressing cells was evident on EMM pH 3.5 media (30 and 37°C) and was not ameliorated by *ssp1* overexpression. Low pH conditions may affect nutritional status in these cells. Failure of CaMKK to activate AMPK [24] in cells with a drop in energy may consequently inhibit processes such as protein synthesis and growth, and affect downstream regulators.

### 3.3. Loss of *ssp1* reduces the restrictive temperature for *cdc25-22*

**Figure 2.**
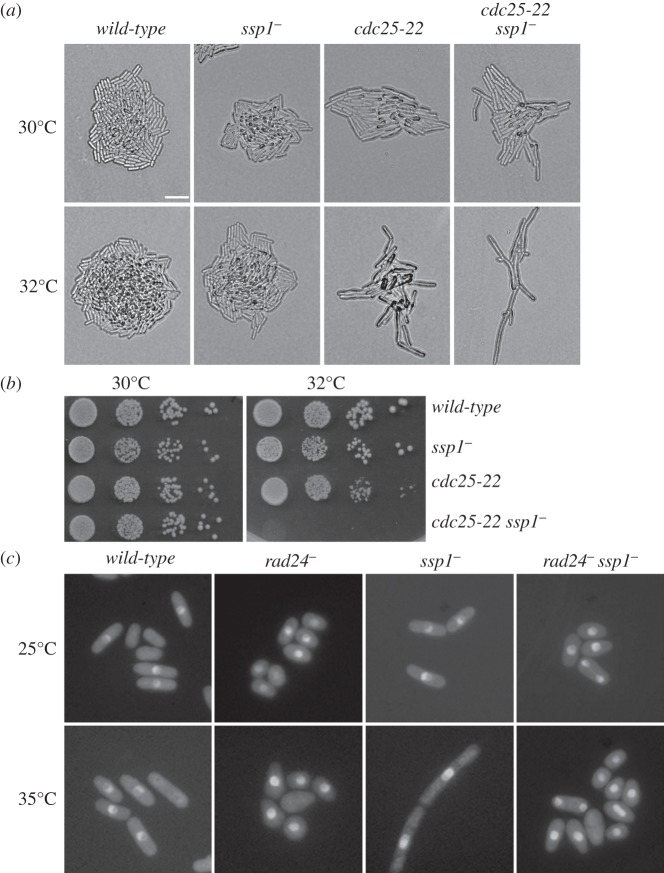
Interaction of *ssp1^−^* with *cdc25-22*. (*a*) Logarithmically growing strains as indicated were streaked onto YEA medium and incubated for 24 h at 30 or 32°C. Bar indicates 10 μm. (*b*) Cells (YEA) were diluted to 10^6^, 10^5^, 10^4^ and 10^3^ cells ml^−1^ and 5 µl spotted onto YEA plates. Cells were incubated for several days at 30 or 32°C. (*c*) Various strains expressing *cdc25*-*GFPint* (YEA, 25°C) were shifted to 35°C for 4 h.

The *cdc25-22^ts^* (Q300) allele at the restrictive temperature (36–36.5°C) arrests at the G_2_/M boundary as very elongated single cells [33,37,38]. The *ssp1^−^* background exacerbates the cell elongation phenotype of *cdc25-22^ts^* at semi-permissive temperatures (32°C) (figure 2*a*,*b*). As *ssp1^−^* cells are sensitive to KCl stress and Cdc25 is exported out of the nucleus within 10 min after KCl stress [39], we investigated the effect of *ssp1^−^* on the nuclear localization of Cdc25. We expressed single-copy, native promoter-integrated *cdc25*-*GFP* (*cdc25-GFPint*) [40] in *rad24^−^ ssp1^−^* (Q4113) and *ssp1^−^* (Q3974) backgrounds at 25°C and shifted to 35°C for 4 h. The nuclear localization of Cdc25-GFP is independent of *ssp1^−^* and *rad24^−^* single and *ssp1^−^ rad24^−^* double gene deletions in both conditions (figure 2*c*), suggesting that the loss of *ssp1* does not interfere with the nuclear localization of Cdc25-GFP.

### 3.4. Deletion of *rad24* overrides growth sensitivity after 0.6 M KCl stress in *ssp1^−^* cells

**Figure 3.**
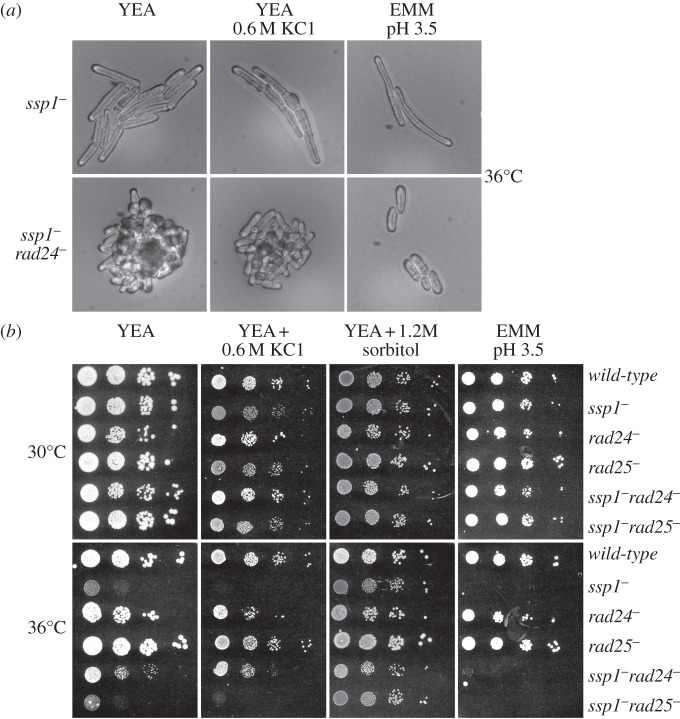
Loss of *rad24* relieves cell-cycle delay and KCl stress sensitivity in *ssp1^−^* cells at 36°C. (*a*) Cells were grown overnight on YEA medium at 30°C, streaked onto YEA, YEA + 0. 6 M KCl or EMM pH 3.5 and incubated at 36°C overnight. (*b*) Logarithmically growing cells in YEA medium were diluted to 10^6^, 10^5^, 10^4^ or 10^3^ cells ml^−1^. Five microlitres of each cell suspension was spotted onto YEA, YEA + 0.6 M KCl and EMM pH 3.5 and incubated at 30 and 36°C for 5 days.

At high temperatures and in the presence of 0.6 M–1.5 M KCl or low pH (3.5), *ssp1*^−^ cells arrest at the G_2_/M boundary (figure 3*a*) [23]. *rad24^−^* alleviates this arrest at high temperatures on YEA and in the presence of KCl. *rad24^−^* is therefore epistatic with respect to *ssp1^−^* cell-cycle arrest following 0.6 M KCl stress at 36°C. Proliferation at pH 3.5 at 36°C is not rescued and small cell size suggests a block to growth (figure 3, also see figure 1). Growth rate in *ssp1^−^* is markedly reduced at high temperatures as indicated by the small colony size in spot tests (figure 3*b*). The response of Ssp1 to 0.6 M KCl stress and to low pH probably occurs through different mechanisms.

### 3.5. At native expression levels Ssp1-GFP localizes to the cell membrane after KCl stress

**Figure 4.**
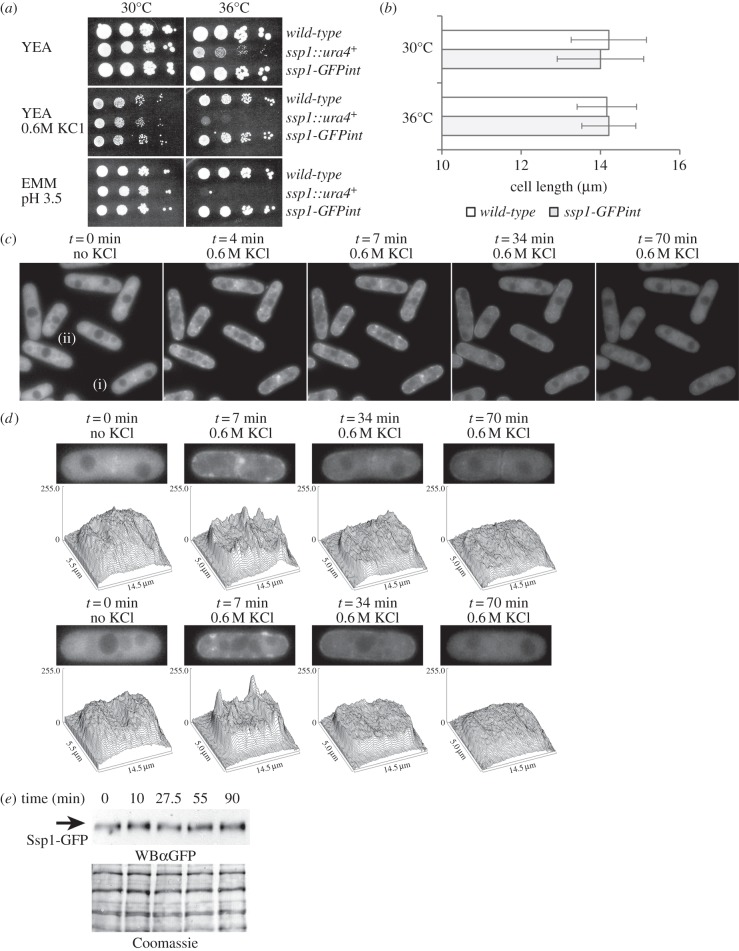
Ssp1-GFP expressed as a chromosomal integrant on its native promoter localizes to the cell membrane following 0.6 M KCl stress. (*a*,*b*) *ssp1*-*GFPint* cells are phenotypically wild-type. (*a*) Cells (YEA, 30°C) were diluted to 10^6^, 10^5^, 10^4^, 10^3^ ml^−1^ and 5 µl of suspension were spotted onto media and incubated for 5 days (30 and 36°C). (*b*) Wild-type and *ssp1-GFPint* cells (YEA, 30°C) were incubated for 4 h at 30 and 36°C. (*c*) *ssp1-GFPint* (YEA, 30°C) cells were analysed in a microfluidic growth chamber supplied with fresh YEA at room temperature. YEA + 0.6 M KCl added at *t*_0_ induced hyperosmotic stress. Cells were imaged 11 times from *t* = 0 to 70 min. Some images were omitted for the sake of brevity. (*d*) Surface fluorescence intensity plots of *ssp1*-*GFPint* cells. Single-plane images of cells from figure 5*c* at *t* = 0 (no stress) and *t* = 7, *t* = 34 and *t* = 70 min (0.6 M KCl stress) were analysed further (surface plot function; ImageJ). (*e*) Ssp1-GFP protein levels after addition of KCl to 0.6 M. Cells were harvested at the indicated times. WBα, western blot with antibody.

GFP-Ssp1 localization was previously examined following strong overexpression (*nmt1*) from a multi-copy plasmid [20,23,41], however variations in cell shape, strength and duration of fluorescence signals in unperturbed and osmotically challenged cells were evident [23]. Cells overexpressing *GFP-ssp1* often accumulate fluorescence at the cell membrane even when unperturbed. We expressed the chromosomal integrant *ssp1-GFPint* (*ssp1::ura4^+^ ssp1-GFP LEU2*) using the *ssp1* native promoter. *ssp1-GFPint* cells grow similarly to wild-type under normal and stress conditions (YEA, YEA + 0.6 M KCl and EMM pH 3.5; 30 and 36°C) (figure 4*a*,*b*). To investigate the pattern and timing of re-localization of Ssp1-GFP at native expression levels following KCl stress *ssp1-GFPint* cells in mid-logarithmic growth were examined in a microfluidic flow chamber (Y2 microfluidic plate, CellASIC), allowing media changes without mechanical perturbation (ONIX Flow Control System, CellASIC). Fluorescence in unperturbed cells expressing *ssp1-GFPint* is largely cytoplasmic. Vacuoles are faintly discernible, suggesting that Ssp1-GFP levels are lower than those in the cytoplasm (figure 4*c*). After switching from YEA to YEA + 0.6 M KCl, cell shapes became more jagged and fission scars became more notable, with a slight decrease in cell volume. Reversal of this cell ‘shrivelling’ with the re-establishment of cell turgor depends upon glycerol synthesis and requires *gpd1^+^* [42]. The nucleus appeared compressed and irregular in shape. Vacuolar areas became more conspicuous, with low fluorescence intensity similar to the nucleus. Within 2–3 min of 0.6 M KCl treatment, small areas of increased Ssp1-GFP fluorescence (foci) in the cytoplasm and near the cell walls and septum appeared. These foci brightened further and by 7 min, additional fluorescent foci along the cell membrane appeared, especially at the cell tips (figure 4*c*,*d*). Localization of Ssp1-GFP at the cell membrane started to decline by 20 min, decreased further by 34 min and was similar to cytoplasmic levels by 70 min. Cells accommodated to the increase in extracellular osmolytes, regaining volume and rod-shaped cell morphology at 23–34 min, indicating induction of *gpd1^+^* and glycerol synthesis [42]. Surface plots of fluorescence intensity of cells at *t* = 0, *t* = 7, *t* = 34 and *t* = 70 min during 0.6 M KCl stress (figure 4*d*) highlights the formation of the small foci of fluorescence intensity in the stressed cells. The conspicuous placement of fluorescent foci, often on opposing sides along the cylindrical portion of the cell suggested that Ssp1-GFP localization to this area takes place not only as distinct foci, but instead as a ring. Although overall fluorescence declined, Ssp1-GFP protein levels stayed constant (figure 4*e*).

**Figure 5.**
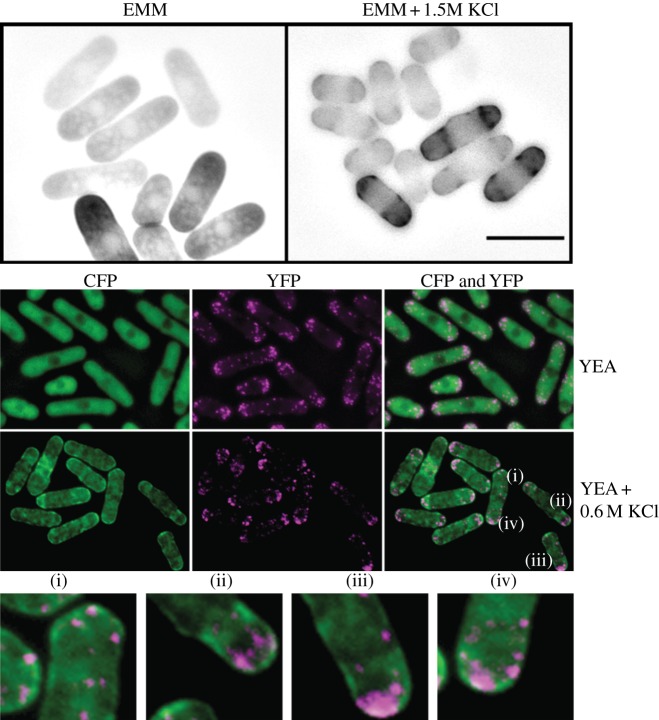
Subcellular localization of Ssp1 and Arp3C. (*a*) GFP-Ssp1 accumulates in a banding pattern near the cell poles after KCl stress. Multi-copy *nmt1-GFP-ssp1* was derepressed for 20 h and hyperosmotic shock induced (EMM + KCl 1.5 M). (*b*) Ssp1-CFP and Arp3C-YFP localization in unperturbed conditions (YEA, 30°C) and after KCl treatment. Cells (YEA, 30°C) expressing single-copy *ssp1-CFPint* were treated with equal amounts of YEA or YEA + 0.6 M KCl. Slidebook software was used to deconvolve Z-stacks of Arp3C-YFP images followed by creation of a projection image also containing single-plane Ssp1-CFP expressed in green for visibility. Scale bar, 10 μm.

Expression of multi-copy *GFP-ssp1* from the *nmt1* promoter (Q4108) facilitated the capture of a multi-image Z-stack. In EMM + 1.5 M KCl (25°C), a band of foci formed around the circumference of the cell (figure 5*a*). This band was not visible in control cells. To determine whether Ssp1 co-localizes with actin patches, we co-expressed single-copy, integrated *ssp1-CFP* and *arp3C*-*YFP* [43] (Q4117) which co-localizes with cortical actin patches. Mid-logarithmic cells (YEA, 25°C) were treated with YEA or YEA + 0.6 M KCl. Projection images of deconvolved Arp3C-YFP (Slidebook; Z-stack) and single Ssp1-CFP images revealed that after 15 min of osmotic stress, the vast majority of actin patches in the cell membrane and/or cell wall area do not co-localize with the accumulated Ssp1-CFP at the cell membrane (figure 5*b*).

### 3.6. Loss of *rad24* or *rad25* does not affect localization of Ssp1-GFP after stress

**Figure 6.**
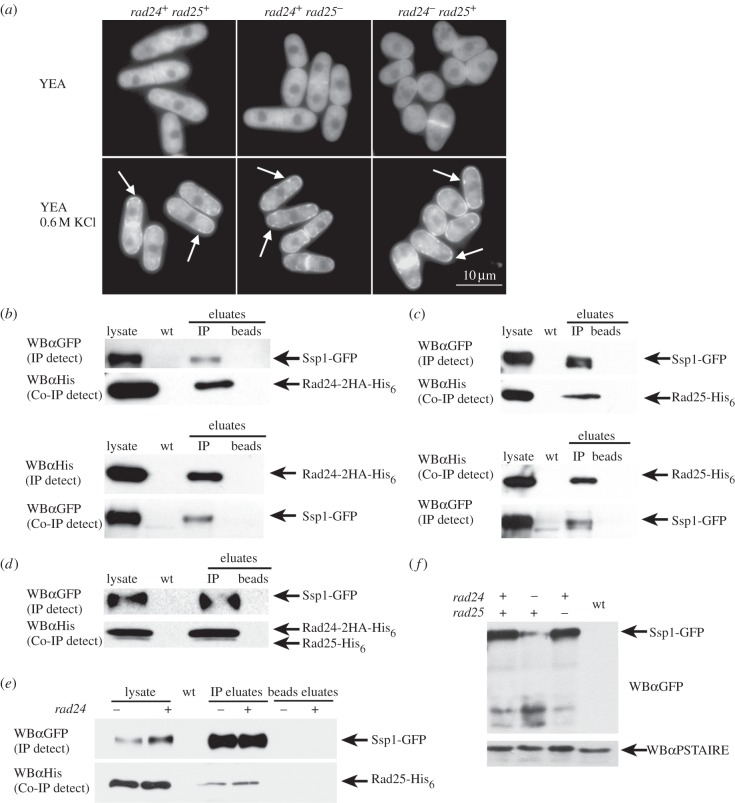
Ssp1-GFP physically interacts with Rad24-2HA-His_6_ and Rad25-His_6_. (*a*) Ssp1-GFP localization in *rad24^−^* and *rad25^−^* cells. Cells (YEA, 30°C) were incubated with pre-warmed YEA, 0.6 M KCl. Fluorescence images were taken prior to and at 15 min after the addition of KCl. (*b–f*) Ssp1-GFP interacts with Rad24-2HA-His_6_ and Rad25-His_6_ *in vivo*. Cells co-expressing Ssp1-GFP and Rad24-2HA-His_6_ protein (*b*), Ssp1-GFP and Rad25-His_6_ protein (*c*), Ssp1-GFP with both Rad24-2HA-His_6_ and Rad25-His_6_ or Ssp1-GFP and Rad25-His_6_ (*rad24^+^* or *rad24^−^*) proteins (*d*) were grown at 30°C in YEA. Aliquots of whole cell lysates used for the immunoprecipitations were loaded (5–15 μg total protein) and Ssp1-GFP, Rad24-2HA-His_6_ and Rad25-His_6_ fusion proteins were directly detected. (*e*) Rad25-His_6_ interacts with Ssp1-GFP in the absence of *rad24*. (*f*) Reduced stability of Ssp1-GFP in the absence of *rad24*. WBα, western blot with antibody.

To investigate how loss of 14-3-3 proteins affects the subcellular localization of Ssp1-GFP, we expressed *ssp1-GFPint* in *rad24^−^* (Q4116) or *rad25^−^* (Q4115) cells. In YEA (30°C), Ssp1-GFP in mid-logarithmic *rad24^−^* or *rad25^−^* cells was cytoplasmic and excluded from the nucleus. Ssp1-GFP accumulated along forming and formed septa, and along the cell membrane in a subset of cells. After osmotic stress (YEA, 0.6 M KCl) Ssp1-GFP promptly localized to areas near the cell membrane, forming foci of fluorescence as in *rad24^+^ rad25^+^* cells (figure 6*a*), indicating that Ssp1-GFP localization to the cell membrane after 0.6 M KCl stress does not require *rad24^−^* or *rad25^−^*.

### 3.7. Ssp1-GFP co-immunoprecipitates with the 14-3-3 proteins Rad24-2HA-His_6_ and Rad25-His_6_

14-3-3 proteins are abundant in all cells, and in budding yeast interact with at least 271 proteins representing approximately 4.4% of the proteome [44] indicating that only a small amount of the total pool of 14-3-3 associates with any one protein at any given time. Only a portion of the total Ssp1-GFP present in the lysate immunoprecipitated in the Rad24-2HA-His_6_ pulldown. To test the relative binding of Rad24 or Rad25 to Ssp1-GFP, protein lysates were prepared from mid-logarithmically growing (YEA, 30°C) cells expressing single-copy *ssp1-GFPint* and either *rad24-2HA-His_6_* (Q4118) or *rad25-His_6_* (Q4119) integrated at their native promoters. Ssp1-GFP co-immunoprecipitates with Rad24 and Rad25 (figure 6*b*,*c*) and a portion of each of the Ssp1 and 14-3-3 pools co-immunoprecipitate.

In budding yeast, the deletion phenotype of the major 14-3-3 isoform BMH1 is complemented by overexpression of the minor isoform BMH2, suggesting that these proteins have similar binding partners [45]. To determine the relative amounts of Rad24 and Rad25 associating with Ssp1, we expressed *ssp1-GFP rad24-2HA-His_6_ rad25*-*His_6_* (Q4120), where Rad24-2HA-His_6_ is distinguishable from Rad25-His_6_ by size owing to small differences in molecular weight as well as the presence of the 2HA. Rad24-2HA-His_6_ was present at approximately fivefold higher levels (ImageJ) compared with Rad25-His_6_ protein in the cell lysate (figure 6*d*) and a similar ratio of Rad24-2HA-His_6_ and Rad25-His_6_ co-precipitated with Ssp1-GFP.

Ssp1-GFP is a highly phosphorylated protein [19]. We can see doublet formation on gels (see immunoprecipitates; figure 6*c*) and can separate these bands by running the SDS-PAGE gel for an extended amount of time. The phosphorylation state of Ssp1-GFP will be discussed below.

### 3.8. Rad25-His_6_ and Ssp1-GFP physically interact in the absence of Rad24

For the previous co-immunoprecipitation studies, Rad24-2HA-His_6_ or Rad25-His_6_ proteins were co-expressed with Ssp1-GFP. We wanted to determine whether Rad25 associates with Ssp1-GFP only as part of a heterodimer with Rad24 or is able to bind Ssp1 as a Rad25–Rad25 homodimer *in vivo*. Immunoprecipitation of Ssp1-GFP and Rad25-His_6_ in *rad24^+^* and *rad24^−^* (Q4121) cells showed that Rad25-His_6_ co-precipitates with Ssp1-GFP in the absence of Rad24 (figure 6*e*). We also found that Ssp1-GFP is less stable in a *rad24^−^* background (figure 6*e*,*f*).

### 3.9. Stress reduces the interaction between Ssp1-GFP and Rad24-2HA-His_6_

**Figure 7.**
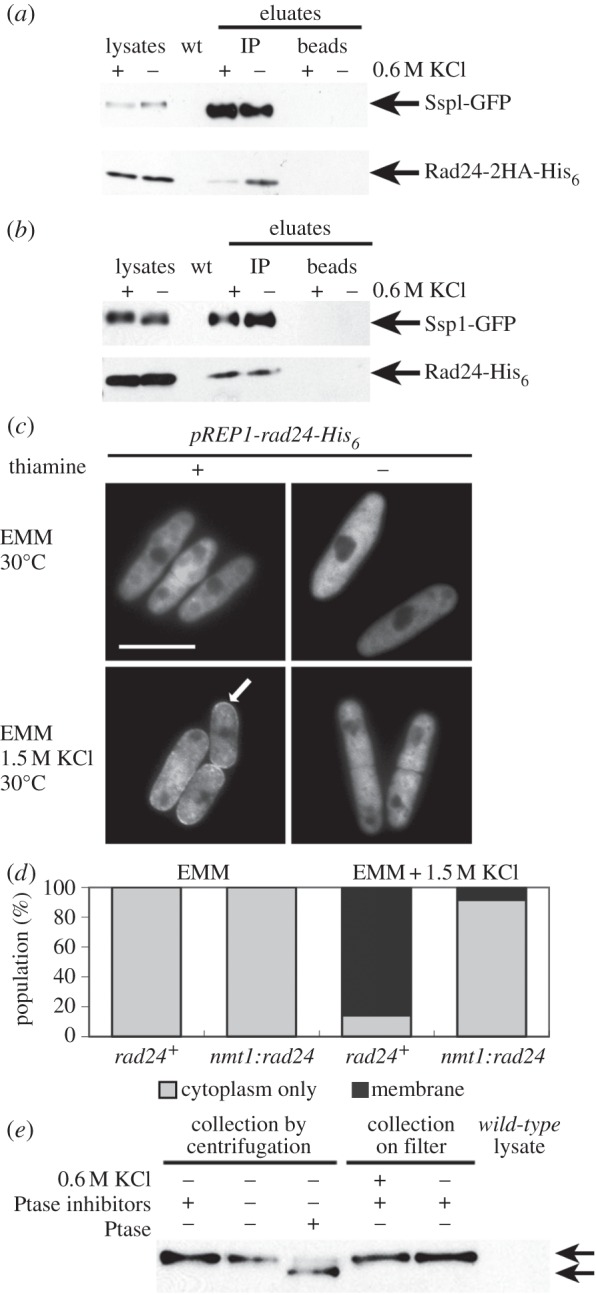
Treatment with 0.6 M KCl for 15 min reduces Rad24-2HA-His_6_ co-immunoprecipitation with Ssp1-GFP. Cells were co-expressing Ssp1-GFP and Rad24-2HA-His_6_ (*a*) or Ssp1-GFP and Rad25-His_6_ (30°C, YEA). YEA + KCl to 0.6 M (30°C) (*b*) was added to aliquots of cells. Ssp1-GFP (5–10 μg), Rad24-2HA-His_6_ (2.5–10 μg) and Rad25-His_6_ (15 μg) were detected in the cell lysates used for the immunoprecipitation. (*c*,*d*) Overexpression of *rad24-His*_6_ reduces Ssp1-GFP cell membrane localization after 0.6 M KCl treatment. (*c*) A plasmid producing Rad24-His_6_ under the control of the *nmt1* promoter was expressed in *ssp1-GFP:kan^R^int* (30°C) in EMM (–thiamine) for 19 h. Images were taken 10–15 min after 1.5 M KCl stress. (*d*) Single-copy *nmt1:GFP-ssp1* was overexpressed in either a *rad24*^+^ background or co-overexpressed with the single-copy integrant *nmt1:rad24*^−^*His_6_* in the absence of thiamine for 20 h (30°C). (*e*) Phosphorylation state of Ssp1-GFP in YEA and YEA + 0.6 M KCl. Cell extracts were prepared in the presence (lane 1) and absence (lane 2) of phosphatase inhibitors. Phosphatase-inhibitor-free Ssp1-GFP extracts (5 μg) were treated with Lambda phosphatase as indicated. Cells were collected on a filter after treatment with YEA or YEA to 0.6 M KCl (30°C). Upper arrow denotes Ssp1-GFP before treatment with Lambda phosphatase.

Harvesting cells by centrifugation at 4°C, followed by washes in ice-cold lysis buffer exposes cells to stressors, including increased gravitational forces and hypoxia. This is manifested by transient increased phosphorylation of MAPK Spc1 [46,47] and Atf1 [46]. Cells undergo a brief cell-cycle delay similar to but shorter than the delay after 0.6 M KCl [23,46] and there is a transient depolarization of actin [46]. Hypoxia in pelleted cells causes activation of hypoxia response genes via Sre1 [48,49]. Moderate thermal downshift (28–15°C) brings about phosphorylation of Spc1 and induction of the stress response genes *ctt1*, *tps1* and *ntp1* [46]. To minimize these stressors, cells were treated with pre-warmed YEA (30°C; ±KCl to 0.6 M KCl) for 15 min, then rapidly chilled to 0°C with frozen, crushed YEA (±0.6 M KCl) preceding centrifugation. Very low temperatures greatly delay Spc1 phosphorylation [46]. Ssp1-GFP localizes to the cell membrane after 15 min of 0.6 M KCl stress and activating phosphorylation of Spc1 MAPK is detected [10]. After KCl treatment approximately 10 times less Rad24-2HA-His_6_ co-immunoprecipitated with Ssp1-GFP in the KCl-treated immunoprecipitate than in the untreated controls (figure 7*a*), however we consistently failed to detect a decrease in the total amount of Rad25-His_6_ protein that co-immunoprecipitated with Ssp1-GFP (figure 7*b*). Ssp1-GFP binds Rad24-2HA-His_6_ and Rad25-His_6_ during unperturbed growth *in vivo*, however bound Rad24-2HA-His_6_ but not Rad25-His_6_ decreases substantially after 0.6 M KCl stress treatment relative to unperturbed conditions *in vivo*. The limitations of immunoblotting do not allow detection of very subtle changes in Rad25-His_6_ binding.

### 3.10. Overexpression of *rad24* diminishes Ssp1-GFP localization to the cell membrane

Loss of either 14-3-3 homologue perturbs MAPK hyperosmolarity stress-dependent signalling in budding yeast [44]. BMH2 physically interacts with the NHA1 antiporter protein at the membrane in a HOG1-independent manner. Loss of BMH1 increases sensitivity to NaCl, KCl and LiCl without affecting plasma membrane potential [45]. In *Schizosaccharomyces pombe*, MAPK activation after osmotic stress leads to phosphorylation of Cdc25 by Srk1, binding of 14-3-3 and Cdc25 nuclear export [14]. Loss of *rad24* or *rad25* does not prevent the stress-dependent localization of Ssp1-GFP to the cell membrane, nor does it increase sensitivity to temperature, low pH or KCl stress (figure 3*a*,*b*). Strong expression of multi-copy *pREP1:rad24-His_6_* in *ssp1-GFPint* cells (Q4124) (EMM, 30°C) impairs Ssp1-GFP accumulation at the membrane after 1 M KCl treatment (figure 7*c*). In *rad24^+^* cells expressing single-copy integrated *GFP-ssp1* under control of the *nmt1* promoter (Q4111), the majority accumulate GFP-Ssp1 at the membrane after KCl stress. This localization pattern is suppressed by co-overexpression of single-copy, integrated *rad24-His_6_* under control of the *nmt1* promoter, where this response is only detected in approximately 12% of the cell population (figure 7*d*).

### 3.11. The phosphorylation state of Ssp1 is not altered after 0.6 M KCl stress

Like all CaMKKs [18], Ssp1 is regulated by phosphorylation. Ssp1 has potential phosphorylation sites at Y58, S59, Y63, T82 and S94, where S59 and S94 were identified in phosphopeptides containing only one phosphorylatable Ser residue [19,50]. Ssp1 is dephosphorylatable *in vitro* [19]. We investigated whether stress response causes changes in Ssp1 phosphorylation. To preserve the phosphorylation state of Ssp1-GFP in unperturbed and KCl-stressed cells, we collected mid-logarithmically growing cells by gentle filtration [35,51] after 15 min treatment with pre-warmed YEA (±KCl to 0.6 M, 30°C). We detected a higher mobility band in extracts treated with Lambda phosphatase (no phosphatase inhibitors; figure 7*e*, lane 3). The faint upper band and strong lower band represent Ssp1-GFP protein in a partially phosphorylated and dephosphorylated state. The lower band is absent in the mock-treated extract (lane 2), confirming that it is not due to proteolysis. There was no bandshift evident in extracts prepared in the presence of phosphatase inhibitors in either perturbed or KCl-treated cells (lanes 4 and 5). Bands corresponded to the position of the phosphorylated bands produced by the control lysates. Ssp1-GFP thus appears to be phosphorylated in both unperturbed (YEA, collected on filter [52] and stressed with 0.6 M KCl) cells *in vivo*. We obtained similar results when lysates were extracted from Ssp1-GFP cells after mild temperature stress (36°C) (not shown). Our results confirm basal phosphorylation of Ssp1.

## 4. Discussion

Regulation of CaMKK occurs through Ca^2+^/CaM binding and inhibitory and activating changes in phosphorylation state. Additional inhibition takes place through 14-3-3 binding [17,32]. Here, we explore the interaction of CaMKK Ssp1 with 14-3-3 and their role in cell-cycle regulation and stress response.

### 4.1. Genetic interaction with 14-3-3 links CaMKK Ssp1 to cell-cycle control machinery

The CaMKK Ssp1 is a mitotic activator [20,23]. Its link to the cell-cycle machinery is supported by suppression of the *ssp1^−^* mitotic delay by *rad24^−^* and the elongation and arrest of *ssp1^−^* cells when overexpressing *rad24*. Mitotic advancement can be interpreted as an additive effect of high levels of CaMKK activity and loss of *rad24* [31]. Human [53] and *Xenopus* [54] 14-3-3 binds to the mitotic inhibitor Wee1 to negatively regulate the cell cycle, through increasing Wee1 half-life, protein levels and kinase activity [53]. Fission yeast 14-3-3 may act similarly on Wee1, where increases in 14-3-3 contribute to G_2_/M delay. Ectopically augmented levels of the mitotic activator Ssp1 presumably titrates out some of the excess Rad24, reducing its effect on other binding partners. *ssp1* overexpression by itself causes mitotic advance indicating a codominant relationship upon overexpression where Ssp1 and Rad24 work independently in an opposing manner. Fission yeast Cdk1 Y15 dephosphorylation by Cdc25 (and Pyp3) phosphatase and phosphorylation by Wee1 (and Mik1) kinase provide positive and negative regulation of cell-cycle progression, respectively [34,55–62]. Wee1 is negatively regulated by phosphorylation through the mitotic activators Cdr1 and Cdr2 kinases [58,63–68]. Ssp1 CaMKK involvement in mitotic control is demonstrated by the reduction of the restrictive temperature of *cdc25-22* by the loss of *ssp1.* The negative additive effect on cell-cycle progression suggests that loss of Ssp1 further inhibits Cdk1, presumably via net increase in Y15 phosphorylation probably through impacting Wee1 kinase activity. Together, these findings support a role for CaMKKs in cell-cycle regulation.

### 4.2. CaMKK Ssp1 does not co-localize with actin patches at the cell membrane

At native expression levels, cytoplasmic Ssp1-GFP accumulates at the cell membrane after perturbation by either 0.6–1.2 M KCl or sorbitol [23]; however *ssp1* is required for growth in the presence of KCl but not sorbitol. Similarly, although MAPK Pmk1 is activated by 1.2 M sorbitol, *pmk1^−^* cells are not sensitive to this hyperosmotic stress [69]. A small pool of Ssp1-GFP accumulates at the cell membrane, while the majority of the protein remains cytoplasmic. This does not support a previous model suggesting that Ssp1 directly localizes actin patches at the cell membrane to support areas of new growth [23]. We show that following hyperosmotic stress GFP-Ssp1 forms a band, while actin patches do not follow this pattern; Arp3C-YFP and Ssp1-CFP do not co-localize. The importance of the compartmentalization of Ssp1 at the membrane is not clear. The *S. pombe* cell wall is most vulnerable to rupture at the extensile tips and is sturdier in the cylindrical portion of the cell [70]. Ssp1 accumulates in areas corresponding to fission scars, which are less vulnerable to damage than the extensile tips [70]. Accumulation at the cell membrane following hyperosmotic stress is transient; however, Ssp1 is required for long-term cell survival under hyperosmotic conditions.

### 4.3. The role of 14-3-3 binding to CaMKK

At least some cytoplasmic Ssp1 is bound to Rad24 and Rad25 in unperturbed cells. After applying hyperosmotic stress, Ssp1 is released from 14-3-3 and Ssp1 accumulates at the cell membrane. Our data also show that Rad24 and Rad25 are dispensable for the translocation of Ssp1-GFP to the cell membrane. This response can be repressed in cells overexpressing Rad24 even when there is an excess of Ssp1-GFP.

14-3-3 proteins commonly act as cytoplasmic anchors, providing negative regulation of proteins through sequestration. In *Drosophila melanogaster*, 14-3-3 binds phosphorylated β-catenin antagonist Chibby (Cby), promoting cytoplasmic sequestration of the β-catenin–14-3-3–Cby complex [71]. In mammalian systems cytoplasmic Bax, a Bcl-2-related protein required for c-Jun NH_2_-terminal kinase-dependent apoptosis, localizes to the mitochondria after stress stimuli, where it induces cytochrome *c* release. 14-3-3-bound Bax remains anchored in the cytoplasm [72]. Upon activation, JNK phosphorylates 14-3-3, Bax is released and translocates to the mitochondria, and apoptosis commences [73]. The Ras/Raf/MEK/ERK-signalling cascade is also regulated by 14-3-3 proteins, which are thought to inhibit activation of plasma membrane-anchored Ras by sequestering its activator Raf-1 in the cytoplasm. This mechanism prevents cascade activation in resting cells [74]. The majority of Ras superfamily G proteins are kept in inactive (GDP-bound) or active (GTP-bound) forms by guanine nucleotide exchange factors and GTPase-activating proteins, respectively. The atypical Rho GTPases Rnd1/2/3 are cell morphology regulators and constitutively bind GTP [75,76]. After phosphorylation by Rock1 kinase or protein kinase Cα (PKCα) [77,78], Rnd3 binds 14-3-3 and translocates from the plasma membrane to the cytoplasm, its function inhibited via sequestration from its site of action [79]. In fission yeast, 14-3-3 proteins also regulate the localization of many proteins. For example, they associate with the primarily cytoplasmic Byr2, preventing binding to Ras1/GTP at the cell membrane during vegetative growth. Loss of 14-3-3 expedites Byr2 translocation [80]. Our data suggest that in fission yeast, 14-3-3 proteins may play a role in the negative regulation of Ssp1 translocation to the cell membrane.

Mammalian 14-3-3 isoforms require only their N-termini to dimerize. Dimerization greatly increases their thermostability and single recombinant isoforms form homodimers even if they function as heterodimers *in vivo* [81,82]. Particular isoforms in 14-3-3 heterodimers allow the interaction of proteins by bringing the two binding partners closer together ([82–84]; reviewed in [85–87]). The minor isoform Rad25, which associates with Ssp1, forms homodimers and binds Ssp1, at least in the absence of Rad24. A reduction in Rad24 binding to Ssp1 after osmotic stress could occur owing to a decrease in Rad24–Rad24 homodimer and/or Rad24–Rad25 heterodimer binding. Conversely, following hyperosmotic stress, Rad25–Rad25 homodimers remain bound to Ssp1-GFP. This suggests an interesting and distinct role for the minor 14-3-3 isoform in CaMKK regulation. Future studies will confirm whether the Rad24-bound pool is preferentially located in any particular part of the cell.

### 4.4. Increased stress sensitivity in cells overexpressing *rad24* is alleviated by co-overexpression of CaMKK

Rad24 is a negative regulator of mitosis after DNA damage [88,89]. Overexpression of *rad24* increases long-term sensitivity to stressors such as KCl and low pH, but sensitivity to high temperature is relieved to some extent by co-overexpression of *ssp1*. Association with 14-3-3 proteins inhibits CaMKK activity in mammalian systems [32,90], thus if Rad24 binding inhibits Ssp1 activity then augmenting levels of 14-3-3 would diminish Ssp1-mediated stress response. Ssp1-FLAG binds 14-3-3, but it is unclear whether this association affects CaMKK activity [19]. 14-3-3 proteins are involved in many pathways [91,92] and substantially increasing the Rad24 pool causes a complex response in terms of stress sensitivity. Both deletion and overexpression of 14-3-3 *BMH1* increase chronological lifespan in nutrient-stressed budding yeast. Cells overexpressing *BMH1* survive longer in the absence of additional stressors, probably because an increase in phosphorylated BMH1 S238 decreases the stress response required for longevity [93].

### 4.5. A role for 14-3-3 in CaMKK turnover

The absence of Rad24 increases Ssp1 turnover, suggesting a role for Rad24 in maintaining Ssp1 protein stability. *rad24^−^* cells do not display hypersensitivity to conditions where *ssp1^−^* cells are unable to proliferate, indicating that Ssp1 protein levels are maintained at sufficient levels. 14-3-3 proteins are involved in protein stabilization both directly and indirectly in other systems, for example by blocking access of ubiquitin ligases (reviewed in [85,86]). In mammalian systems, association with 14-3-3 prevents Wee1 degradation by masking a degradation motif required for normal Wee1 turnover [94]. Budding yeast 14-3-3 homologues BMH1, BMH2 and ACM1 form a stable complex with the APC/C activator CDH1/CDC20, keeping it inactived by acting as an APC pseudosubstrate [95,96].

Further studies will determine whether the interaction of 14-3-3 and Ssp1 has a direct or indirect effect on CaMKK catalytic function and whether 14-3-3 proteins play a role in the negative regulation of Ssp1 translocation to the cell membrane after stress.

## 5. Material and methods

### 5.1. Plasmid construction and chromosomal integration

All DNA amplification was performed by PCR with Expand High Fidelity *Taq* Polymerase (Roche). T4 ligase and restriction enzymes used were from Promega. For primers, see table 2.

**Table 2.** List of oligonucleotides.

primer	sequence
*Ssp1intforw*	*5′ gggggctgcagttgagttagcctactggattatcttat 3′*
*Ssp1intrev*	*5′ ggggggtcgacgaattagttggtgtgaaggaatgctct 3′*
*Rad25HisForward*	*5′ gggggctgcagcattgcagtagaa 3′*
*Rad25HisReverse*	*5′ ggggggtcgactcagtggtgatgatggtgatgagctttaacagtgtcagtcg 3′*
*rad24OPforw*	*5′ actgtcatatgtctactacttctcgtgaagatgct 3′*
*rad24OPrev*	*5′ actgtgtcgactcagtggtgatgatggtgatgtgcgtccgccttgggctc 3′*
*SFrad24-f*	*5′ gggggcatatgtctactacttctcgtgaagatgct 3′*
*SFrad24-r*	*5′ ggggggtcgactttgcgtccgccttgggcgca 3′*
*KanShortForwTM*	*5′ tctaactaccttttaca 3′*
*KanShortRevTM*	*5′ tctattatgaatttcat 3′*
*KrnfFW-17*	*5′ agcttgtgatattgacg 3′*
*KrnfRV-17*	*5′ agcttagctacaaatcc 3′*

*pssp1-GFPint* and *pssp1-CFPint*: the *ssp1* gene (+1000 bp upstream sequence) was amplified from *S. pombe* genomic DNA [97] (primers *Ssp1intforw* and *Ssp1intrev*) adding *Pst*I and *Sal*I restriction sites. The *nmt1* promoter was excised from the *pREP1-GFP* and *pREP1-CFP* vectors with *Pst*I and *Sal*I [41,98,99] and the *ssp1* +1000 bp fragment was inserted, generating *pssp1-GFPint* and *pssp1-CFPint.* The plasmids were integrated into *ssp1::ura4^+^ leu1-32 ura4-D18* and stable integrants were identified and tested by out-crossing to a *ura4-D18 leu1-32* strain. Strains were tested for their ability to rescue the *ssp1^−^* phenotype, and normal subcellular localization was confirmed (see Results section).

*pREP1-rad24-His_6_*, *pREP2-rad24-His_6_ and pREP1-rad24-GFP*: the *rad24* ORF was amplified with primers *rad24OPforw* and *rad24OPrev*, adding a C-terminal His_6_ tag, *Nde*I and *Sal*I restriction sites or the primers *SFrad24-f* and *SFrad24-r*, adding *Nde*I and *Sal*I restriction sites, respectively. The fragments were ligated into *pREP1*, *pREP2* [41,98] and *pREP1-GFP* plasmids [99] forming *pREP1-rad24-His_6_*, *pREP1-rad24-His_6_* and *pREP1-rad24-GFP*. Plasmids rescued *rad24::ura4^+^*. A single copy of *pREP2-rad24-His_6_* was integrated into a *leu1-32 ura4-D18* strain.

*Integration of nmt1:GFP-ssp1*: a single copy of the plasmid *pIR2-22*, containing *GFP*-*ssp1* under control of the *nmt1* promoter (*pIR2-22*) [23] was integrated into *leu1*-*32 ura4*-*D18*.

### 5.2. Targeted replacement of *LEU2* and *ura4* with *kanMX6* in *ssp1-GFPint* and *rad25::ura4^+^*

A *kanMX6* cassette with 80 bp sequence homology to *LEU2* at the 5′ and 3′ ends was generated by PCR amplification using primers *KanShortForwTM* and *KanShortRevTM* and the *pGEM-T* (Promega) vector containing *kanMX6*. The *kanMX6* cassette was transformed into *ssp1-GFPint* and plated on YEA (+0.1 mg ml^−1^ G418; Gibco) [100,101]. A *kanMX6* cassette was amplified with *KrnfFW-1*7 and *KrnfRV-17* primers having homology to *URA4* at the 5′ and 3′ ends and transformed into *rad25::ura^+^ ura4-D18*, producing *rad25::kanMX6 ura4-D18*.

### 5.3. Protein lysates

Lysates were prepared at 4°C unless otherwise indicated. Mid-logarithmic growth phase cells were harvested by centrifugation (5 min, 1876.9*g*), washed in ice-cold stop buffer [97], collected again by pulse centrifugation (13 051*g*) and frozen on dry ice. Lysis by mechanical disruption with glass beads (0.5 mm, BioSpec) and a bead beater (MiniBeadBeater-8 Cell Disruptor, BioSpec) in lysis buffer [102] or modified SUME buffer (1% SDS, 8 M urea, 10 mM MOPS pH 6.8, 10 mM EDTA, 50 mM NaF, 1 mM NaVO_4_; [103]) supplemented with complete protease inhibitor (EDTA-free, Roche) was alternated with rest periods in an ice-water slurry (0°C). Lysates were cleared by centrifugation (13 051*g*) and protein concentration determined by Bio-Rad protein assay. Protein extracts were boiled in Laemmli buffer (200 mM Tris–HCl pH 6.8, 8% SDS, 40% glycerol, 3.34% (v/v) 2-mercaptoethanol, 0.01% bromophenol blue).

### 5.4. KCl treatment

Mid-logarithmic growth phase cells (1000–3000 ml, YEA, 30°C) were treated for 15 min with warm (30°C) YEA (control) or YEA + KCl to 0.6 M. Cells were chilled to 0°C within 1 min by the addition of frozen, crushed YEA or YEA + 0.6 M KCl and immediate immersion of flask into an ice-water/ethanol slurry.

### 5.5. Immunoprecipitation

Lysate preparation and immunoprecipitation were performed at 4°C. Cell cultures (YEA, 30°C) were harvested by centrifugation (9927.3*g*) and washed once in 10 ml ice-cold HB buffer [97] (pH 7.4; containing 2 mM DTT and Roche complete protease inhibitor (EDTA free), washed again and followed by mechanical disruption in ice-cold HB buffer. Lysates were centrifuged (35 4406*g*; 40 min) and incubated with 100 µl bed-volume Sepharose G beads (4 Fast Flow, GE Healthcare) for 1 h to remove non-specific binding proteins. Lysates were incubated overnight with 15 µl rabbit anti-GFP polyclonal serum (Invitrogen) or 15 µl mouse anti-His_6_ antibody (Roche) on a rotator. Control lysates did not contain antibodies. Lysates were incubated with 100 µl bed-volume Protein G Sepharose beads for 2 h. Beads were washed extensively with HB buffer and protein complexes eluted with 0.2 M glycine (pH 2.2) for 15 min and neutralized by addition of saturated Tris (pH 10). Supernatants were boiled with Laemmli buffer and analysed by immunoblotting.

### 5.6. Immunoblotting

Extracts and immunoprecipitates were subjected to SDS-PAGE and transferred to a polyvinylidene difluoride (PVDF) membrane (Perkin Elmer). Immunoprecipitated Ssp1-GFP protein was detected with monoclonal anti-GFP antibody (1 : 1000) (Roche). Co-immunoprecipitated Ssp1-GFP protein was detected with polyclonal anti-GFP serum (1 : 1000) (Invitrogen). Immunoprecipitated and co-immunoprecipitated Rad24-2HA-His_6_ and Rad25-His_6_ proteins were detected with monoclonal anti-His_6_ antibody (1 : 1000) (Roche; Genscript). Bands were visualized with goat anti-mouse or goat anti-rabbit HRP-conjugated secondary antibody (1 : 2000) (Santa Cruz Biotechnology) and luminol-based ECL reagent (Perkin Elmer).

### 5.7. Reprobing of polyvinylidene difluoride membranes

PVDF membranes were stripped as described [104] and reprobed with polyclonal anti-PSTAIRE antibody (1 : 500) (Upstate Biotechnology).

### 5.8. Phosphatase treatments

Mid-logarithmic phase cells were treated with 30°C YEA or YEA + 0.6 M KCl for 15 min. To preserve phosphorylation state, cells were collected on microfibre filters (934-AH; Whatman) and washed with 5 ml ice-cold stop buffer [97] lysed in lysis buffer [97] (buffers contained 15 mM pNPP and 60 mM *β*-glycerophosphate). Cells collected by centrifugation were washed (150 mM NaCl, 1 mM EDTA, 1 mM PMSF) and lysed in phosphatase-inhibitor-free lysis buffer (modified from [97]) or washed and lysed with phosphatase-inhibitor-enriched buffers. Phosphatase-inhibitor-free protein (5 μg) was treated with 800 units of Lambda Protein Phosphatase (NEB) for 30 min at 30°C. Mock treatments did not contain phosphatase.

### 5.9. Microscopy

Images were captured by a high performance CCD (Cooke SensiCam) camera on a Leitz DMRB fluorescence microscope or a high performance CCD Hamamatsu Orca-ER camera on a Zeiss AxioImager.Z1 fluorescence microscope. Slidebook image analysis software (Intelligent Image Innovations) was used to perform cell measurements and to analyse Z-stacks.

## Funding statement

This study was financially supported with the assistance of the Natural Sciences and Engineering Research Council, National Cancer Institute of Canada and Canadian Institutes of Health Research.

## References

[1] Causton HC 2001 Remodeling of yeast genome expression in response to environmental changes. Mol. Biol. Cell 12, 323–337. (doi:10.1091/mbc.12.2.323) 11179418 10.1091/mbc.12.2.323 PMC30946

[2] Gasch AP Spellman PT Kao CM Carmel-Harel O Eisen MB Storz G Botstein D Brown PO 2000 Genomic expression programs in the response of yeast cells to environmental changes. Mol. Biol. Cell 11, 4241–4257. (doi:10.1091/mbc.11.12.4241) 11102521 10.1091/mbc.11.12.4241 PMC15070

[3] O'Rourke SM Herskowitz I O'Shea EK 2002 Yeast go the whole HOG for the hyperosmotic response. Trends Genet. 18, 405–412. (doi:10.1016/S0168-9525(02)02723-3) 12142009 10.1016/s0168-9525(02)02723-3

[4] Gustin MC Albertyn J Alexander M Davenport K 1998 MAP kinase pathways in the yeast *Saccharomyces cerevisiae*. Microbiol. Mol. Biol. Rev. 62, 1264–1300. 9841672 10.1128/mmbr.62.4.1264-1300.1998 PMC98946

[5] Chen D Toone WM Mata J Lyne R Burns G Kivinen K Brazma A Jones N Bahler J 2003 Global transcriptional responses of fission yeast to environmental stress. Mol. Biol. Cell 14, 214–229. (doi:10.1091/mbc.E02-08-0499) 12529438 10.1091/mbc.E02-08-0499 PMC140239

[6] Shieh JC Wilkinson MG Buck V Morgan BA Makino K Millar JB 1997 The Mcs4 response regulator coordinately controls the stress-activated Wak1-Wis1-Sty1 MAP kinase pathway and fission yeast cell cycle. Genes Dev. 11, 1008–1022. (doi:10.1101/gad.11.8.1008) 9136929 10.1101/gad.11.8.1008

[7] Samejima I Mackie S Fantes PA 1997 Multiple modes of activation of the stress-responsive MAP kinase pathway in fission yeast. EMBO J. 16, 6162–6170. (doi:10.1093/emboj/16.20.6162) 9321395 10.1093/emboj/16.20.6162 PMC1326300

[8] Warbrick E Fantes PA 1991 The wis1 protein kinase is a dosage-dependent regulator of mitosis in *Schizosaccharomyces pombe*. EMBO J. 10, 4291–4299. 1756736 10.1002/j.1460-2075.1991.tb05007.x PMC453182

[9] Kato T Okazaki K Murakami H Stettler S Fantes PA Okayama H 1996 Stress signal, mediated by a Hogl-like MAP kinase, controls sexual development in fission yeast. FEBS Lett. 378, 207–212. (doi:10.1016/0014-5793(95)01442-X) 8557102 10.1016/0014-5793(95)01442-x

[10] Degols G Shiozaki K Russell P 1996 Activation and regulation of the Spc1 stress-activated protein kinase in *Schizosaccharomyces pombe*. Mol. Cell. Biol. 16, 2870–2877. 8649397 10.1128/mcb.16.6.2870 PMC231280

[11] Shiozaki K Russell P 1996 Conjugation, meiosis, and the osmotic stress response are regulated by Spc1 kinase through Atf1 transcription factor in fission yeast. Genes Dev. 10, 2276–2288. (doi:10.1101/gad.10.18.2276) 8824587 10.1101/gad.10.18.2276

[12] Blázquez MA Stucka R Feldmann H Gancedo C 1994 Trehalose-6-P synthase is dispensable for growth on glucose but not for spore germination in *Schizosaccharomyces pombe*. J. Bacteriol. 176, 3895–3902. 8021171 10.1128/jb.176.13.3895-3902.1994 PMC205586

[13] Gaits F Degols G Shiozaki K Russell P 1998 Phosphorylation and association with the transcription factor Atf1 regulate localization of Spc1/Sty1 stress-activated kinase in fission yeast. Genes Dev. 12, 1464–1473. (doi:10.1101/gad.12.10.1464) 9585506 10.1101/gad.12.10.1464 PMC316836

[14] López-Avilés S Grande M González M Helgesen AL Alemany V Sanchez-Piris M Bachs O Millar JBA Aligue R 2005 Inactivation of the Cdc25 phosphatase by the stress-activated Srk1 kinase in fission yeast. Mol. Cell 17, 49–59. (doi:10.1016/j.molcel.2004.11.043) 15629716 10.1016/j.molcel.2004.11.043

[15] Millar JB Buck V Wilkinson MG 1995 Pyp1 and Pyp2 PTPases dephosphorylate an osmosensing MAP kinase controlling cell size at division in fission yeast. Genes Dev. 9, 2117–2130. (doi:10.1101/gad.9.17.2117) 7657164 10.1101/gad.9.17.2117

[16] Shiozaki K Russell P 1995 Cell-cycle control linked to extracellular environment by MAP kinase pathway in fission yeast. Nature 378, 739–743. (doi:10.1038/378739a0) 7501024 10.1038/378739a0

[17] Racioppi L Means AR 2012 Calcium/calmodulin-dependent protein kinase kinase 2: roles in signaling and pathophysiology. J. Biol. Chem. 278, 31 658–31 665. (doi:10.1074/jbc.R112.356485) 10.1074/jbc.R112.356485 PMC3442500 22778263

[18] Skelding KA Rostas JA 2012 The role of molecular regulation and targeting in regulating calcium/calmodulin stimulated protein kinases. Adv. Exp. Med. Biol. 740, 703–730. (doi:10.1007/978-94-007-2888-2_31) 22453966 10.1007/978-94-007-2888-2_31

[19] Hanyu Y 2009 *Schizosaccharomyces pombe* cell division cycle under limited glucose requires Ssp1 kinase, the putative CaMKK, and Sds23, a PP2A-related phosphatase inhibitor. Genes Cells 14, 539–554. (doi:10.1111/j.1365-2443.2009.01290.x) 19371376 10.1111/j.1365-2443.2009.01290.x

[20] Matsusaka T Hirata D Yanagida M Toda T 1995 A novel protein kinase gene *ssp1^+^* is required for alteration of growth polarity and actin localization in fission yeast. EMBO J. 14, 3325–3338. 7628434 10.1002/j.1460-2075.1995.tb07339.x PMC394400

[21] Shimanuki M Kinoshita N Ohkura H Yoshida T Toda T Yanagida M 1993 Isolation and characterization of the fission yeast protein phosphatase gene *ppe1*+ involved in cell shape control and mitosis. Mol. Biol. Cell 4, 303–313. (doi:10.1091/mbc.4.3.303) 8387356 10.1091/mbc.4.3.303 PMC300928

[22] Toda T Niwa H Nemoto T Dhut S Eddison M Matsusaka T Yanagida M Hirata D 1996 The fission yeast *sts5^+^* gene is required for maintenance of growth polarity and functionally interacts with protein kinase C and an osmosensing MAP-kinase pathway. J. Cell. Sci. 109, 2331–2342. 8886983 10.1242/jcs.109.9.2331

[23] Rupeš I Jia Z Young PG 1999 Ssp1 promotes actin depolymerization and is involved in stress response and new end take-off control in fission yeast. Mol. Biol. Cell 10, 1495–1510. (doi:10.1091/mbc.10.5.1495) 10233158 10.1091/mbc.10.5.1495 PMC25317

[24] Valbuena N Moreno S 2012 AMPK phosphorylation by Ssp1 is required for proper sexual differentiation in fission yeast. J. Cell. Sci. 125, 2655–2664. (doi:10.1242/jcs.098533) 22375066 10.1242/jcs.098533

[25] Matsuzawa T Fujita Y Tohda H Takegawa K 2012 Snf1-like protein kinase Ssp2 regulates glucose derepression in *Schizosaccharomyces pombe*. Eukaryot. Cell 11, 159–167. (doi:10.1128/EC.05268-11) 22140232 10.1128/EC.05268-11 PMC3272901

[26] Hardie DG 2008 AMPK and raptor: matching cell growth to energy supply. Mol. Cell 30, 263–265. (doi:10.1016/j.molcel.2008.04.012) 18471972 10.1016/j.molcel.2008.04.012

[27] Hong SP Leiper FC Woods A Carling D Carlson M 2003 Activation of yeast Snf1 and mammalian AMP-activated protein kinase by upstream kinases. Proc. Natl Acad. Sci. USA 100, 8839–8843. (doi:10.1073/pnas.1533136100) 12847291 10.1073/pnas.1533136100 PMC166400

[28] Sutherland CM Hawley SA McCartney RR Leech A Stark MJ Schmidt MC Hardie DG 2003 Elm1p is one of three upstream kinases for the *Saccharomyces cerevisiae* SNF1 complex. Curr. Biol. 13, 1299–1305. (doi:10.1016/S0960-9822(03)00459-7) 12906789 10.1016/s0960-9822(03)00459-7

[29] Lee YJ Jeschke GR Roelants FM Thorner J Turk BE 2012 Reciprocal phosphorylation of yeast glycerol-3-phosphate dehydrogenases in adaptation to distinct types of stress. Mol. Cell. Biol. 32, 4705–4717. (doi:10.1128/MCB.00897-12) 22988299 10.1128/MCB.00897-12 PMC3486180

[30] Robertson AM Hagan IM 2008 Stress-regulated kinase pathways in the recovery of tip growth and microtubule dynamics following osmotic stress in *S. pombe*. J. Cell. Sci. 121, 4055–4068. (doi:10.1242/jcs.034488) 19033386 10.1242/jcs.034488

[31] Ford J Al-Khodairy F Fotou E Sheldrick K Griffiths D Carr A 1994 14-3-3 protein homologs required for the DNA damage checkpoint in fission yeast. Science 265, 533–535. (doi:10.1126/science.8036497) 8036497 10.1126/science.8036497

[32] Ichimura T Taoka M Hozumi Y Goto K Tokumitsu H 2008 14-3-3 proteins directly regulate Ca^2+^/calmodulin-dependent protein kinase kinase alpha through phosphorylation-dependent multisite binding. FEBS Lett. 582, 661–665. (doi:10.1016/j.febslet.2008.01.037) 18242179 10.1016/j.febslet.2008.01.037

[33] Russell P Nurse P 1986 *Cdc25^+^* functions as an inducer in the mitotic control of fission yeast. Cell 45, 145–153. (doi:10.1016/0092-8674(86)90546-5) 3955656 10.1016/0092-8674(86)90546-5

[34] Nurse P 1975 Genetic control of cell size at cell division in yeast. Nature 256, 547–551. (doi:10.1038/256547a0) 1165770 10.1038/256547a0

[35] Fantes PA Nurse P 1978 Control of the timing of cell division in fission yeast: cell size mutants reveal a second control pathway. Exp. Cell Res. 115, 317–329. (doi:10.1016/0014-4827(78)90286-0) 689088 10.1016/0014-4827(78)90286-0

[36] O'Connell MJ Raleigh JM Verkade HM Nurse P 1997 Chk1 is a wee1 kinase in the G2 DNA damage checkpoint inhibiting cdc2 by Y15 phosphorylation. EMBO J. 16, 545–554. (doi:10.1093/emboj/16.3.545) 9034337 10.1093/emboj/16.3.545 PMC1169658

[37] Nurse P Thuriaux P Nasmyth K 1976 Genetic control of the cell division cycle in the fission yeast *Schizosaccharomyces pombe*. Mol. Gen. Genet. 146, 167–178. (doi:10.1007/BF00268085) 958201 10.1007/BF00268085

[38] Fantes P 1979 Epistatic gene interactions in the control of division in fission yeast. Nature 279, 428–430. (doi:10.1038/279428a0) 16068179 10.1038/279428a0

[39] López-Aviles S Lambea E Moldon A Grande M Fajardo A Rodriguez-Gabriel MA Hidalgo E Aligue R 2008 Activation of Srk1 by the mitogen-activated protein kinase Sty1/Spc1 precedes its dissociation from the kinase and signals its degradation. Mol. Biol. Cell 19, 1670–1679. (doi:10.1091/mbc.E07-07-0639) 18272791 10.1091/mbc.E07-07-0639 PMC2291412

[40] Chua G Lingner C Frazer C Young PG 2002 The sal3^+^ gene encodes an importin-beta implicated in the nuclear import of Cdc25 in *Schizosaccharomyces pombe*. Genetics 162, 689–703. 12399381 10.1093/genetics/162.2.689 PMC1462273

[41] Basi G Schmid E Maundrell K 1993 TATA box mutations in the *Schizosaccharomyces pombe nmt1* promoter affect transcription efficiency but not the transcription start point or thiamine repressibility. Gene 123, 131–136. (doi:10.1016/0378-1119(93)90552-E) 8422997 10.1016/0378-1119(93)90552-e

[42] Minc N Boudaoud A Chang F 2009 Mechanical forces of fission yeast growth. Curr. Biol. 19, 1096–1101. (doi:10.1016/j.cub.2009.05.031) 19500986 10.1016/j.cub.2009.05.031 PMC2790036

[43] Nolen BJ Pollard TD 2008 Structure and biochemical properties of fission yeast Arp2/3 complex lacking the Arp2 subunit. J. Biol. Chem. 283, 26 490–26 498. (doi:10.1074/jbc.M802607200) 10.1074/jbc.M802607200 PMC2546537 18640983

[44] Kakiuchi K 2007 Proteomic analysis of in *vivo* 14-3-3 interactions in the yeast *Saccharomyces cerevisiae*. Biochemistry 46, 7781–7792. (doi:10.1021/bi700501t) 17559233 10.1021/bi700501t

[45] Zahradka J van Heusden GP Sychrova H 2012 Yeast 14-3-3 proteins participate in the regulation of cell cation homeostasis via interaction with Nha1 alkali-metal-cation/proton antiporter. Biochim. Biophys. Acta 1820, 849–858. (doi:10.1016/j.bbagen.2012.03.013) 22484491 10.1016/j.bbagen.2012.03.013

[46] Soto T Nunez A Madrid M Vicente J Gacto M Cansado J 2007 Transduction of centrifugation-induced gravity forces through mitogen-activated protein kinase pathways in the fission yeast *Schizosaccharomyces pombe*. Microbiology 153, 1519–1529. (doi:10.1099/mic.0.2006/004283-0) 17464066 10.1099/mic.0.2006/004283-0

[47] Shiozaki K Shiozaki M Russell P 1998 Heat stress activates fission yeast Spc1/StyI MAPK by a MEKK-independent mechanism. Mol. Biol. Cell 9, 1339–1349. (doi:10.1091/mbc.9.6.1339) 9614178 10.1091/mbc.9.6.1339 PMC25354

[48] Todd BL Stewart EV Burg JS Hughes AL Espenshade PJ 2006 Sterol regulatory element binding protein is a principal regulator of anaerobic gene expression in fission yeast. Mol. Cell. Biol. 26, 2817–2831. (doi:10.1128/MCB.26.7.2817-2831.2006) 16537923 10.1128/MCB.26.7.2817-2831.2006 PMC1430309

[49] Hughes AL Todd BL Espenshade PJ 2005 SREBP pathway responds to sterols and functions as an oxygen sensor in fission yeast. Cell 120, 831–842. (doi:10.1016/j.cell.2005.01.012) 15797383 10.1016/j.cell.2005.01.012

[50] Wilson-Grady JT Villén J Gygi SP 2008 Phosphoproteome analysis of fission yeast. J. Proteome Res. 7, 1088–1097. (doi:10.1021/pr7006335) 18257517 10.1021/pr7006335

[51] Young PG Fantes PA 1987 *Schizosaccharomyces pombe* mutants affected in their division response to starvation. J. Cell. Sci. 88, 295–304. 3448096 10.1242/jcs.88.3.295

[52] Fantes P Nurse P 1977 Control of cell size at division in fission yeast by a growth-modulated size control over nuclear division. Exp. Cell Res. 107, 377–386. (doi:10.1016/0014-4827(77)90359-7) 872891 10.1016/0014-4827(77)90359-7

[53] Rothblum-Oviatt CJ Ryan CE Piwnica-Worms H 2001 14-3-3 binding regulates catalytic activity of human Wee1 kinase. Cell Growth Differ. 12, 581–589. 11751453

[54] Lee J Kumagai A Dunphy WG 2001 Positive regulation of Wee1 by Chk1 and 14-3-3 proteins. Mol. Biol. Cell 12, 551–563. (doi:10.1091/mbc.12.3.551) 11251070 10.1091/mbc.12.3.551 PMC30963

[55] Nurse P Bissett Y 1981 Gene required in G1 for commitment to cell cycle and in G2 for control of mitosis in fission yeast. Nature 292, 558–560. (doi:10.1038/292558a0) 7254352 10.1038/292558a0

[56] Thuriaux P Nurse P Carter B 1978 Mutants altered in the control co-ordinating cell division with cell growth in the fission yeast *Schizosaccharomyces pombe*. Mol. Gen. Genet. 161, 215–220. 672898 10.1007/BF00274190

[57] Piggott JR Rai R Carter BL 1982 A bifunctional gene product involved in two phases of the yeast cell cycle. Nature 298, 391–393. (doi:10.1038/298391a0) 7045699 10.1038/298391a0

[58] Russell P Nurse P 1987 Negative regulation of mitosis by *wee1^+^*, a gene encoding a protein kinase homolog. Cell 49, 559–567. (doi:10.1016/0092-8674(87)90458-2) 3032459 10.1016/0092-8674(87)90458-2

[59] Gould KL Nurse P 1989 Tyrosine phosphorylation of the fission yeast cdc2^+^ protein kinase regulates entry into mitosis. Nature 342, 39–45. (doi:10.1038/342039a0) 2682257 10.1038/342039a0

[60] Featherstone C Russell P 1991 Fission yeast p107wee1 mitotic inhibitor is a tyrosine/serine kinase. Nature 349, 808–811. (doi:10.1038/349808a0) 1825699 10.1038/349808a0

[61] Lundgren K Walworth N Booher R Dembski M Kirschner M Beach D 1991 Mik1 and Wee1 cooperate in the inhibitory tyrosine phosphorylation of Cdc2. Cell 64, 1111–1122. (doi:10.1016/0092-8674(91)90266-2) 1706223 10.1016/0092-8674(91)90266-2

[62] Parker LL Atherton-Fessler S Piwnica-Worms H 1992 P107wee1 is a dual-specificity kinase that phosphorylates P34cdc2 on tyrosine 15. Proc. Natl Acad. Sci. USA 89, 2917–2921. (doi:10.1073/pnas.89.7.2917) 1372994 10.1073/pnas.89.7.2917 PMC48774

[63] Feilotter H Nurse P Young PG 1991 Genetic and molecular analysis of cdr1/nim1 in *Schizosaccharomyces pombe*. Genetics 127, 309–318. 2004705 10.1093/genetics/127.2.309 PMC1204358

[64] Coleman TR Tang Z Dunphy WG 1993 Negative regulation of the wee1 protein kinase by direct action of the nim1/cdr1 mitotic inducer. Cell 72, 919–929. (doi:10.1016/0092-8674(93)90580-J) 7681363 10.1016/0092-8674(93)90580-j

[65] Parker LL Walter SA Young PG Piwnica-Worms H 1993 Phosphorylation and inactivation of the mitotic inhibitor Wee1 by the nim1/cdr1 kinase. Nature 363, 736–738. (doi:10.1038/363736a0) 8515817 10.1038/363736a0

[66] Wu L Russell P 1997 Roles of Wee1 and Nim1 protein kinases in regulating the switch from mitotic division to sexual development in *Schizosaccharomyces pombe*. Mol. Cell. Biol. 17, 10–17. 8972180 10.1128/mcb.17.1.10 PMC231724

[67] Breeding CS Hudson J Balasubramanian MK Hemmingsen SM Young PG Gould KL 1998 The *cdr2*^+^ gene encodes a regulator of G2/M progression and cytokinesis in *Schizosaccharomyces pombe*. Mol. Biol. Cell 9, 3399–3415. (doi:10.1091/mbc.9.12.3399) 9843577 10.1091/mbc.9.12.3399 PMC25645

[68] Kanoh J Russell P 1998 The protein kinase Cdr2, related to Nim1/Cdr1 mitotic inducer, regulates the onset of mitosis in fission yeast. Mol. Biol. Cell 9, 3321–3334. (doi:10.1091/mbc.9.12.3321) 9843572 10.1091/mbc.9.12.3321 PMC25629

[69] Madrid M Soto T Khong HK Franco A Vicente J Perez P Gacto M Cansado J 2006 Stress-induced response, localization, and regulation of the Pmk1 cell integrity pathway in *Schizosaccharomyces pombe*. J. Biol. Chem. 281, 2033–2043. (doi:10.1074/jbc.M506467200) 16291757 10.1074/jbc.M506467200

[70] Piombo S Calleja GB Yoo BY Johnson BF 1998 Ruptured fission yeast walls: structural discontinuities related to the cell cycle. Cell Biochem. Biophys. 29, 263–279. (doi:10.1007/BF02737898) 9868582 10.1007/BF02737898

[71] Li FQ Mofunanya A Harris K Takemaru K 2008 Chibby cooperates with 14-3-3 to regulate beta-catenin subcellular distribution and signaling activity. J. Cell Biol. 181, 1141–1154. (doi:10.1083/jcb.200709091) 18573912 10.1083/jcb.200709091 PMC2442201

[72] Nomura M Shimizu S Sugiyama T Narita M Ito T Matsuda H Tsujimoto Y 2003 14-3-3 interacts directly with and negatively regulates pro-apoptotic Bax. J. Biol. Chem. 278, 2058–2065. (doi:10.1074/jbc.M207880200) 12426317 10.1074/jbc.M207880200

[73] Tsuruta F Sunayama J Mori Y Hattori S Shimizu S Tsujimoto Y Yoshioka K Masuyama N Gotoh Y 2004 JNK promotes Bax translocation to mitochondria through phosphorylation of 14-3-3 proteins. EMBO J. 23, 1889–1899. (doi:10.1038/sj.emboj.7600194) 15071501 10.1038/sj.emboj.7600194 PMC394248

[74] Light Y Paterson H Marais R 2002 14-3-3 antagonizes Ras-mediated Raf-1 recruitment to the plasma membrane to maintain signaling fidelity. Mol. Cell. Biol. 22, 4984–4996. (doi:10.1128/MCB.22.14.4984-4996.2002) 12077328 10.1128/MCB.22.14.4984-4996.2002 PMC139778

[75] Riou P Villalonga P Ridley AJ 2010 Rnd proteins: multifunctional regulators of the cytoskeleton and cell cycle progression. Bioessays 32, 986–992. (doi:10.1002/bies.201000060) 20836090 10.1002/bies.201000060

[76] Foster R Hu KQ Lu Y Nolan KM Thissen J Settleman J 1996 Identification of a novel human Rho protein with unusual properties: GTPase deficiency and *in vivo* farnesylation. Mol. Cell. Biol. 16, 2689–2699. 8649376 10.1128/mcb.16.6.2689 PMC231259

[77] Madigan JP 2009 Regulation of Rnd3 localization and function by protein kinase C alpha-mediated phosphorylation. Biochem. J. 424, 153–161. (doi:10.1042/BJ20082377) 19723022 10.1042/BJ20082377 PMC2868966

[78] Riento K Totty N Villalonga P Garg R Guasch R Ridley AJ 2005 RhoE function is regulated by ROCK I-mediated phosphorylation. EMBO J. 24, 1170–1180. (doi:10.1038/sj.emboj.7600612) 15775972 10.1038/sj.emboj.7600612 PMC556412

[79] Riou P 2013 14-3-3 proteins interact with a hybrid prenyl-phosphorylation motif to inhibit G proteins. Cell 153, 640–653. (doi:10.1016/j.cell.2013.03.044) 23622247 10.1016/j.cell.2013.03.044 PMC3690454

[80] Ozoe F Kurokawa R Kobayashi Y Jeong HT Tanaka K Sen K Nakagawa T Matsuda H Kawamukai M 2002 The 14-3-3 proteins Rad24 and Rad25 negatively regulate Byr2 by affecting its localization in *Schizosaccharomyces pombe*. Mol. Cell. Biol. 22, 7105–7119. (doi:10.1128/MCB.22.20.7105-7119.2002) 12242289 10.1128/MCB.22.20.7105-7119.2002 PMC139824

[81] Aitken A Jones D Soneji Y Howell S 1995 14-3-3 proteins: biological function and domain structure. Biochem. Soc. Trans. 23, 605–611. 8566426 10.1042/bst0230605

[82] Jones DH Ley S Aitken A 1995 Isoforms of 14-3-3 protein can form homo-and heterodimers *in vivo* and *in vitro*: implications for function as adapter proteins. FEBS Lett. 368, 55–58. (doi:10.1016/0014-5793(95)00598-4) 7615088 10.1016/0014-5793(95)00598-4

[83] Shen YH Godlewski J Bronisz A Zhu J Comb MJ Avruch J Tzivion G 2003 Significance of 14-3-3 self-dimerization for phosphorylation-dependent target binding. Mol. Biol. Cell 14, 4721–4733. (doi:10.1091/mbc.E02-12-0821) 14551260 10.1091/mbc.E02-12-0821 PMC266786

[84] Tzivion G Shen YH Zhu J 2001 14-3-3 proteins: bringing new definitions to scaffolding. Oncogene 20, 6331–6338. (doi:10.1038/sj.onc.1204777) 11607836 10.1038/sj.onc.1204777

[85] Aitken A 2002 Functional specificity in 14-3-3 isoform interactions through dimer formation and phosphorylation: chromosome location of mammalian isoforms and variants. Plant Mol. Biol. 50, 993–1010. (doi:10.1023/A:1021261931561) 12516867 10.1023/a:1021261931561

[86] Aitken A Baxter H Dubois T Clokie S Mackie S Mitchell K Peden A Zemlickova E 2002 Specificity of 14-3-3 isoform dimer interactions and phosphorylation. Biochem. Soc. Trans. 30, 351–360. (doi:10.1042/BST0300351) 12196094 10.1042/bst0300351

[87] Sluchanko NN Gusev NB 2010 14-3-3 proteins and regulation of cytoskeleton. Biochemistry 75, 1528–1546. (doi:10.1134/S0006297910130031) 21417993 10.1134/s0006297910130031

[88] Tallada VA Daga RR Palomeque C Garzon A Jimenez J 2002 Genome-wide search of *Schizosaccharomyces pombe* genes causing overexpression-mediated cell cycle defects. Yeast 19, 1139–1151. (doi:10.1002/yea.902) 12237855 10.1002/yea.902

[89] Zeng Y Piwnica-Worms H 1999 DNA damage and replication checkpoints in fission yeast require nuclear exclusion of the Cdc25 phosphatase via 14-3-3 binding. Mol. Cell. Biol. 19, 7410–7419. 10523629 10.1128/mcb.19.11.7410 PMC84734

[90] Davare MA Saneyoshi T Guire ES Nygaard SC Soderling TR 2004 Inhibition of calcium/calmodulin-dependent protein kinase kinase by protein 14-3-3. J. Biol. Chem. 279, 52 191–52 199. (doi:10.1074/jbc.M409873200) 10.1074/jbc.M409873200 15469938

[91] van Hemert MJ Steensma HY van Heusden GP 2001 14-3-3 proteins: key regulators of cell division, signalling and apoptosis. Bioessays 23, 936–946. (doi:10.1002/bies.1134) 11598960 10.1002/bies.1134

[92] van Heusden GP Steensma HY 2006 Yeast 14-3-3 proteins. Yeast 23, 159–171. (doi:10.1002/yea.1338) 16498703 10.1002/yea.1338

[93] Wang LY Shiozaki K 2006 The fission yeast stress MAPK cascade regulates the *pmp3+* gene that encodes a highly conserved plasma membrane protein. FEBS Lett. 580, 2409–2413. (doi:10.1016/j.febslet.2006.03.065) 16603158 10.1016/j.febslet.2006.03.065

[94] Wang Y Jacobs C Hook KE Duan H Booher RN Sun Y 2000 Binding of 14-3-3beta to the carboxyl terminus of Wee1 increases Wee1 stability, kinase activity, and G2-M cell population. Cell Growth Differ. 11, 211–219. 10775038

[95] Martinez JS Jeong DE Choi E Billings BM Hall MC 2006 Acm1 is a negative regulator of the CDH1-dependent anaphase-promoting complex/cyclosome in budding yeast. Mol. Cell. Biol. 26, 9162–9176. (doi:10.1128/MCB.00603-06) 17030612 10.1128/MCB.00603-06 PMC1698549

[96] Dial JM Petrotchenko EV Borchers CH 2007 Inhibition of APCCdh1 activity by Cdh1/Acm1/Bmh1 ternary complex formation. J. Biol. Chem. 282, 5237–5248. (doi:10.1074/jbc.M606589200) 17178718 10.1074/jbc.M606589200

[97] Moreno S Klar A Nurse P 1991 Molecular genetic analysis of fission yeast *Schizosaccharomyces pombe*. Meth. Enzymol. 194, 795–823. (doi:10.1016/0076-6879(91)94059-L) 2005825 10.1016/0076-6879(91)94059-l

[98] Maundrell K 1993 Thiamine-repressible expression vectors pREP and pRIP for fission yeast. Gene 123, 127–130. (doi:10.1016/0378-1119(93)90551-D) 8422996 10.1016/0378-1119(93)90551-d

[99] Taricani L Tejada ML Young PG 2002 The fission yeast ES2 homologue, Bis1, interacts with the Ish1 stress-responsive nuclear envelope protein. J. Biol. Chem. 277, 10 562–10 572. (doi:10.1074/jbc.M110686200) 10.1074/jbc.M110686200 11751918

[100] Chua G Taricani L Stangle W Young PG 2000 Insertional mutagenesis based on illegitimate recombination in *Schizosaccharomyces pombe*. Nucleic Acids Res. 28, e53 (doi:10.1093/nar/28.11.e53) 10871352 10.1093/nar/28.11.e53 PMC102638

[101] Bahler J Nurse P 2001 Fission yeast Pom1p kinase activity is cell cycle regulated and essential for cellular symmetry during growth and division. EMBO J. 20, 1064–1073. (doi:10.1093/emboj/20.5.1064) 11230130 10.1093/emboj/20.5.1064 PMC145493

[102] Taricani L Feilotter HE Weaver C Young PG 2001 Expression of hsp16 in response to nucleotide depletion is regulated via the spc1 MAPK pathway in *Schizosaccharomyces pombe*. Nucleic Acids Res. 29, 3030–3040. (doi:10.1093/nar/29.14.3030) 11452028 10.1093/nar/29.14.3030 PMC55794

[103] Gardner RG Nelson ZW Gottschling DE 2005 Degradation-mediated protein quality control in the nucleus. Cell 120, 803–815. (doi:10.1016/j.cell.2005.01.016) 15797381 10.1016/j.cell.2005.01.016

[104] Hakki M Geballe AP 2005 Double-stranded RNA binding by human cytomegalovirus pTRS1. J. Virol. 79, 7311–7318. (doi:10.1128/JVI.79.12.7311-7318.2005) 15919885 10.1128/JVI.79.12.7311-7318.2005 PMC1143672

